# Estrogen regulation of testicular function

**DOI:** 10.1186/1477-7827-3-51

**Published:** 2005-09-27

**Authors:** Benson T Akingbemi

**Affiliations:** 1 Department of Anatomy, Physiology and Pharmacology, College of Veterinary Medicine, Auburn University, Auburn, AL 36849, USA

## Abstract

Evidence supporting a role for estrogen in male reproductive tract development and function has been collected from rodents and humans.  These studies fall into three categories: i) localization of aromatase and the target protein for estrogen (ER-alpha and ER-beta) in tissues of the reproductive tract; ii) analysis of testicular phenotypes in transgenic mice deficient in aromatase, ER-alpha and/or ER-beta gene; and, iii) investigation of the effects of environmental chemicals on male reproduction.  Estrogen is thought to have a regulatory role in the testis because estrogen biosynthesis occurs in testicular cells and the absence of ERs caused adverse effects on spermatogenesis and steroidogenesis.  Moreover, several chemicals that are present in the environment, designated xenoestrogens because they have the ability to bind and activate ERs, are known to affect testicular gene expression.  However, studies of estrogen action are confounded by a number of factors, including the inability to dissociate estrogen-induced activity in the hypothalamus and pituitary from action occurring directly in the testis and expression of more than one ER subtype in estrogen-sensitive tissues.  Use of tissue-specific knockout animals and administration of antiestrogens and/or aromatase inhibitors in vivo may generate additional data to advance our understanding of estrogen and estrogen receptor biology in the developing and mature testis.

## Introduction

The testis consists of two compartments: seminiferous tubules and intertubular tissue, which forms the interstitium. Seminiferous tubules are lined by layers of germ cells in various stages of development (spermatogonia, spermatocytes, spermatids, spermatozoa) and supporting Sertoli cells. The interstitium consists of loose connective tissue, blood and lymphatic vessels, and various cell types, including Leydig cells, fibroblasts, macrophages and leukocytes. Leydig cells are the predominant source of the male sex steroid hormone testosterone. However, recent observations challenge the dogma that the male phenotype is maintained solely by testosterone binding to its protein target, i.e., the androgen receptor. Growing public concerns that exposures to environmental chemicals with estrogenic activity may impact human reproductive health have focused attention on the role of estrogen in male reproductive health [1]. The aromatization of C19 androgens, i.e., testosterone and androstenedione, is a key step in estrogen (E2) biosynthesis and is catalyzed by the aromatase enzyme, which is a product of the *CYP*19 gene [2]. The serum levels of E2 measure about 40 pg/mL in male rats [3], and ranges between 20 and 40 pg/mL in men [4]. Evidence from several studies indicates that aromatase, ERα and ERβ are encoded by separate genes but are co-expressed with androgen receptors in the male reproductive tract [2,3]. In consonance with localization studies, mice which have targeted deletion of the aromatase gene, ERα and/or ERβ showed altered testicular morphology and derangements of spermatogenesis [5-7], and exposures of laboratory species and wildlife to estrogenic chemicals were found to cause abnormalities of the reproductive tract [8].

Although the present review is focused on direct estrogen action in the testis, estrogen regulation may occur indirectly by changes caused in the hypothalamus and pituitary. Gonadal steroids act on the hypothalamus to affect GnRH pulses, and at the pituitary level to regulate gonadotropin (FSH and LH) secretion. FSH and LH are the primary tropic hormones that regulate testicular function. Indeed, FSH receptors are expressed only in Sertoli cells, and Leydig cells are the only binding sites for LH in the testis. In contrast, ERs have a more diversified pattern of expression. There is conclusive evidence showing that ERα and ERβ are present in several hypothalamic nuclei and in pituitary gonadotropes, indicating that estrogen regulates the hypothalamus-pituitary axis [9,10]. For example, E2 treatment of a mouse gonadotroph cell line (LβT2) increased LH secretion and, following co-incubation with GnRH, increased LHβ mRNA levels [11]. Furthermore, the presence of estrogen-response-elements (EREs) on the promoter region of the β-subunit of the LH gene has been reported, implying that estrogen regulation of LH secretion occurs directly at the level of the LHβ gene [12,13]. There is also evidence that ERα can be transcriptionally activated in gonadotrope cells in an estrogen-independent manner, through the GnRH receptor and signaling via protein kinase C (PKC) and mitogen-activated protein kinase (MAPK) pathways [14]. Together, these observations demonstrate that estrogen regulation of testicular function is also mediated indirectly by changes occurring in the hypothalamus and pituitary.

## Estrogen Receptors

**Figure 1:**
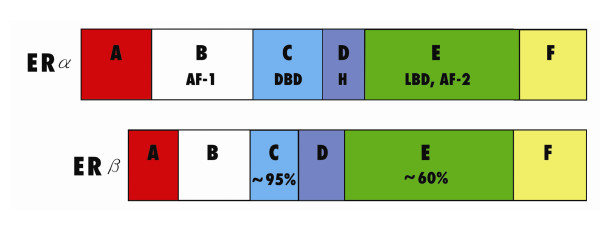
An illustration of the structure of the estrogen receptor. The NH_2 _terminal consists of the A/B domains, the C domain forms the DNA-binding domain (DBD) while domains D/E/F constitute the ligand-binding domain (LBD). The AF-1 and AF-2 activation units are part of the DNA-binding and ligand-binding domains, respectively. The two ER subtypes, ERα and ERβ, are almost identical in the DNA-binding domain (~95% homology) but differ in the ligand-binding domain (about 60% homology). Differences in the ligand-binding domain are responsible in part for ligand-specificity, and the ratio of ERα and ERβ is a critical determinant of cellular response to endogenous estrogen and other ER agonists and antagonists.

ERs are members of the steroid/thyroid hormone super family of nuclear receptors, which share a common structural architecture, and consist of three independent but interacting functional domains: the N-terminal or A/B domains, the C or DNA-binding domain, and the D/E/F or ligand-binding domain (Fig 1). Binding of a ligand to the ER causes a series of downstream events, including receptor dimerization, receptor-DNA interactions mediated by EREs present in the promoter region of target genes, recruitment of and interaction with transcription factors, and the formation of a preinitiation complex. Ligand-receptor interactions ultimately cause changes in target gene expression [15]. The N-terminal domain of nuclear receptors encodes an activation function called AF-1, which mediates protein-protein interactions to induce transcriptional activity. It is thought that this domain is highly active in ERα-mediated stimulation of reporter gene expression from a variety of ERE-constructs but its activity in the ERβ is limited [16]. On the other hand, the C-terminal or ligand-binding domain contains the AF-2 interacting surface that mediates ligand binding and receptor dimerization to stimulate transcriptional activity [17]. Thus, AF-1 and AF-2 are both involved in mediating the transcriptional activation functions of ERs.

Although there is a high degree of homology in the DNA-binding domains of ERα and ERβ (about 95%), only a partial homology exists in the ligand-binding domain (~60%) [18]. Differences in ligand binding, in association with other factors, have the effect of altering the pattern of ER-mediated transcriptional activity. For example, some agonists bind both ER subtypes with the same affinity while others preferentially bind to ERα or ERβ [19-21]. There is general agreement that ERs function as dimers, and co-expression of ERα and ERβ in the same cell causes the formation of homodimers (ERα/ERα and ERβ/ERβ) or heterodimers (ERα/ERβ), which affect ligand-specificity. The interactions between ERs and EREs are complicated by other factors, including the ability of ERβ to modulate ERα transcriptional activity and recruitment of several protein co-activators and repressors by both ER subtypes. Therefore, the relative amounts of ERα and ERβ in a given tissue are key determinants of cellular responses to estrogen and other ER agonists and antagonists [22]. Moreover, ER and other steroid receptors have the ability to mediate biological effects through non-transcriptional mechanisms mediated by protein-protein interactions occurring between ERs and growth factors e.g., IGF-1 and EGF [23,24]. Furthermore, there is growing evidence for the presence of a small pool of ERs localized to the plasma membrane. For example, BSA-conjugated E2, which is unable to gain entry into the cytosol and acts at the plasma membrane, decreased testicular androgen production *in vitro *[25]. Membrane ER is thought to signal mainly by coupling to GTP-activating proteins and through pathways involving second messengers (e.g., calcium) and kinase cascades [26]. The integration of several pathways implies that estrogen action in any particular tissue and organ is the result of activities mediated by genomic and non-genomic pathways although the physiological significance of specific pathways in the testis remains to be elucidated [27].

## I. Localization of aromatase and ERs

Data describing aromatase activity and ER expression in reproductive tissues were collected using a combination of techniques: binding assays, immunohistochemistry, *in situ *hybridization, reverse transcriptase-polymerase chain reaction (RT-PCR), and RNase protection assays. In spite of the large body of information derived from these studies, localization studies have shortcomings that limit data interpretation regarding ER expression in specific tissues. For example, binding assays do not distinguish between ERα and ERβ while *in situ *hybridization studies measure mRNA levels but do not determine whether mRNA is translated to protein. Similarly, immunocytochemistry lacked specificity for either ER subtype. However, the availability of antibodies directed against ERα, and much later for ERβ, allowing for discrimination between ER subtypes in subsequent studies has generated substantial information on ER biology.

During fetal development in the rodent, aromatase is expressed in Sertoli cells and Leydig cells but not in spermatogonia. On the other hand, aromatase has been localized to virtually all cell types in the adult testis, including Leydig cells, Sertoli cells, spermatocytes, spermatids and spermatozoa [6,28,29]. Cellular expression of aromatase is age-dependent in the postnatal rat, occurring predominantly in Sertoli cells and germ cells of the prepubertal testis (up to 21 days of age) and in Leydig cells after this period [30]. Taken together, the bulk of data collected from the rodent testis point to a general pattern of ERα expression in Leydig cells and peritubular myoid cells and ERβ in germ cells [10,31-33]. However, ERβ was localized to adult Leydig cells in the mouse [34], and both ERα and ERβ were found to be present in rat fetal and adult Leydig cells [35]. Similarly, ERα and ERβ were immunolocalized to Leydig cells in pubertal rats although treatment with the pure antiestrogen ICI 182,780 abolished ERα, but not ERβ, protein [36]. Consistent with ER expression in diverse cell types in the testis, it is not surprising that administration of ER agonists and antagonists or targeted deletion of the aromatase gene and ERs caused derangements in germ cell development and testicular steroidogenesis.

Unlike in rodents, aromatase activity and estrogen biosynthesis occur mostly in adipose tissue in men, and the testis synthesizes only 10–25% of E2 in circulation [37]. Early studies showed that prenatal exposures to the synthetic estrogen diethylstilbestrol (DES) caused male reproductive tract abnormalities in mice [38] and men [39]. In agreement with these observations, ERα and ERβ were localized to the human testis, and the presence of two variants of ERβ, designated ERβ1 and ERβ2, has been clearly demonstrated [40]. Although ERβ mRNA levels were 3-fold greater than ERα, both were expressed in the testis beginning from 16 weeks of gestation [41]. ERβ1 was more widely expressed in Sertoli cells, germ cells and Leydig cells while ERβ2 mRNA and protein were restricted to spermatogonia [42]. In the adult testis, both ERα and ERβ are expressed in spermatocytes, elongating spermatids, Sertoli cells and Leydig cells [43,44]. Other studies have demonstrated the presence of ERα in spermatids and mature spermatozoa [45], ERβ in all germ cells [46,47], and the absence of ERα in Leydig cells [48]. ERβ1 appears to be expressed at high levels in pachytene spermatocytes and round spermatids but much less so in Sertoli cells and spermatogonia whereas expression of ERβ2 is high in Sertoli cells and spermatogonia and is reduced in spermatocytes [49,50]. Although the physiological significance of ERβ isoforms in the human testis remains to be clarified, it has been suggested that ERβ2 forms heterodimers with ERα thereby attenuating its transcriptional activity; however, it lacks the ability to bind endogenous E2 or recruit cofactors via the AF-2 domain [51].

## II. Transgenic mouse studies

Reports of testicular anomalies in men with naturally occurring mutations in the aromatase gene and in individuals lacking a functional ERα, including undescended testis, decreased sperm production, and altered endocrine profiles, reinforced the view that estrogen action is a requirement for normal testicular function [52-55]. Thus, development of knockout or transgenic mice with disruption of molecules related to reproduction and hormone action, e.g., mice with targeted deletion of the aromatase gene (ARKO), ERα (αERKO), ERβ (βERKO) and both ER subtypes (αβERKO), has contributed immensely to our understanding of reproductive endocrinology [56]. A major difference between these lines of mutant mice is that ARKO mice adequately express ERα and ERβ protein and do not make endogenous E2 whereas ER knockout mice are able to synthesize E2 but lack either ERα and/or ERβ protein. Therefore, a major caveat in these studies is the inadvertent removal of estrogen priming of extragonadal tissues during development. In this regard, there is a possibility that absence of endogenous E2 and/or ER-mediated activity during tissue differentiation in the hypothalamus and pituitary jeopardizes developmental maturation of regulatory pathways in the HPT axis.

The spectrum of testicular anomalies exhibited by transgenic mice deficient in E2 biosynthesis and ER protein is summarized in Table 1. ARKO mice have enlarged sex accessory organ weights presumably as a result of elevated serum testosterone levels and enhanced androgen action, and show disturbances of spermatogenesis, which is associated with increased apoptosis of developing germ cells [6,7]. However, the results of fertility assessment in ARKO mice have been rather inconsistent, sexual function being impaired in one line of mice and not in the other; these differences are thought to be due to the amounts of residual aromatase gene products in mutant mice [7,57]. In contrast to the lack of E2, overexpression of the aromatase gene and enhanced E2 production in mice induced cryptorchidism or undescended testis, spermatogenic arrest, Leydig cell hyperplasia, and decreased serum FSH and testosterone levels. Disruption of spermatogenesis was associated with decreased FSH levels while increased exposures to E2 induce Leydig cell hyperplasia [58]. Progressive degeneration of testicular tissue, dilation of the seminiferous tubules, and sexual behavioral problems are typical findings in αERKO mice [5]. Disruption of spermatogenesis has been attributed to fluid retention, which causes pressure atrophy of the seminiferous epithelium [59].

**Table 1:** Testicular function in mice deficient in estrogen biosynthesis or estrogen receptor protein

	Spermatogenesis	Steroidogenesis	Fertility	References
ARKO^a^	Affected	Affected	Affected	6,7
αERKO^b^	Affected	Affected	Affected	5
βERKO^c^	Normal	Normal	Normal	66
αβERKO^d^	Affected	Affected	Affected	64

The obvious differences in the phenotypes of ARKO and αERKO mice, as were determined in early studies, implied that ERα is not the sole mediator of estrogen action and that another ER protein may be present in testicular cells. These speculations were confirmed by cloning of ERβ in the rat prostate and ovary [60]. Subsequently, the bulk of experimental evidence shows that ERβ regulates germ cell development. For example, ERα inactivation had no effect on the number of Sertoli cells and spermatogonia whereas ERβ inactivation increased the number of spermatogonia by more than 50% in neonatal mice [61]. However, it is surprising that in spite of the evidence for ERβ regulation of mitosis in spermatogonia, which serve as stem cells for the process of spermatogenesis, disturbances of sperm production were not evident in βERKO mice. On the other hand, the presence of ERα in Sertoli cells has not been demonstrated. Paradoxically, spermatogenic arrest occurs in αERKO mice, which have ERβ protein. These observations suggest that testicular cells regulate Sertoli cell support of germ cell development through unidentified ERα-mediated mechanisms. This line of thinking is supported by data from experiments in which germ cells were transplanted from donor males homozygous for the mutation ERα^-/- ^to testes of wild-type ERα^+/+ ^recipient mice depleted of germ cells. When mated to wild-type females, the recipients sired offspring heterozygous for the mutation ERα^+/- ^but retained the coat-color marker of the ERα^-/- ^donor mice. This finding confirmed that somatic cells in the testis, but not germ cells, have a requirement for ERα in order to support the process of spermatogenesis [62,63]. In contrast to the αERKO, βERKO males retain full fertility but tend to show increased incidence of prostate hyperplasia with advancing age [64]. Perhaps not unexpectedly, male αβERKO mice are infertile, which is likely due to ERα deficiency because these effects are absent in βERKO mice [65,66].

**Figure 2:**
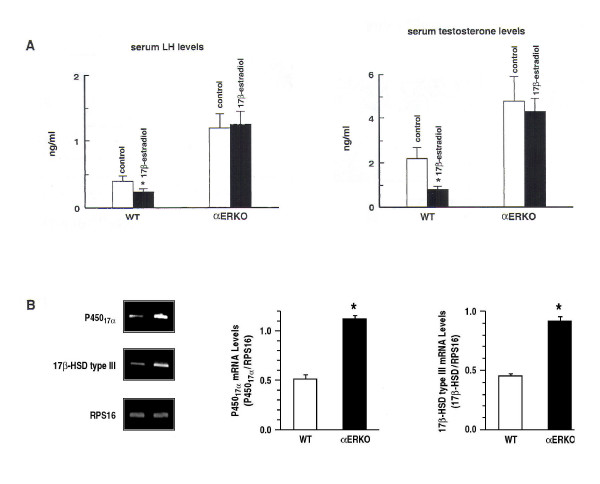
Estrogen regulates testicular steroidogenesis, acting via ERα because administration of an estrogenic chemical, estradiol benzoate, suppressed pituitary LH secretion and serum testosterone levels in wild-type but not αERKO mice (ref. 67)(A). ERα deficiency enhanced gene expression for cytochrome P450 hydroxylase/17α lyase and 17β-hydroxysteroid dehydrogenase type III in αERKO Leydig cells compared to wildtype (WT) (B), indicating that ERα regulates androgen biosynthesis by mediating changes in steroidogenic enzyme activity (ref. 67). *Copyright 2003, The Endocrine Society.*

Alteration of the endocrine profile is a consistent finding in transgenic mice with targeted deletion of aromatase gene or ERs (Table 2). For example, serum LH levels were elevated in adult ARKO mice [6] while serum testosterone concentrations, though were increased at 12–14 wk of age, were comparable in wild-type and mutant mice [7]. Similarly, the concentrations of serum testosterone, LH, and FSH were increased in αERKO males compared to their wild-type littermates [5,67]. The changes in serum gonadotropin levels presumably result from alleviation of estrogen feedback regulation on the hypothalamus-pituitary axis. The regulation of testicular steroidogenesis appears to be mediated primarily by ERα because changes in serum steroid hormone levels seen in the αERKO are absent in βERKO mice. Moreover, administration of a synthetic estrogen, estradiol benzoate, reduced serum LH and testosterone levels in wild-type but not αERKO mice (Fig. 2A), and treatment with the pure antiestrogen ICI 182,780 decreased androgen biosynthesis in wild-type but not αERKO Leydig cells [67]. The differences in androgen biosynthesis between αERKO and wild-type Leydig cells were associated with changes in steroidogenic enzyme activity because ERα deficiency enhanced gene expression for cytochrome P450 hydroxylase/17α lyase and 17β-hydroxysteroid dehydrogenase type III; these enzymes take part in reactions involved in the conversion of the steroid substrate cholesterol to testosterone (Fig. 2B). Consistent with these observations, a recent report showed that DES decreased testosterone production of wild-type fetal and neonatal testes but not ERα^-^/^- ^[68].

**Table 2:** Endocrine profiles of mice deficient in estrogen biosynthesis or estrogen receptor protein

	FSH	LH	Testosterone	17β-estradiol	References
ARKO^a^	Elevated	Elevated	Elevated	Absent	6,7
αERKO^b^	ND	Elevated	Elevated	ND	5,67
βERKO^c^	Normal	Normal	Normal	ND	66
αβERKO^d^	ND	Elevated	Elevated	ND	64

ND, Not determined.

## III. Studies of xenoestrogens

Although there had been long standing evidence that male reproductive tract development is subject to estrogen action [39,69], scientific attention to the role of estrogen in reproductive activity was highlighted only recently by public concerns that exposures to environmental chemicals may adversely affect the endocrine and reproductive systems. Exposures of laboratory animals and wildlife to high levels of estrogenic chemicals resulted in a number of abnormalities, including reduced gonad size, feminization of genetic males, and low sperm count and quality. In this regard, estrogenic activity has been attributed to a diverse array of steroidal and non steroidal compounds, including industrial chemicals (e.g., polychlorobiphenyls, alkyphenols, pesticides (e.g., DDT derivatives, methoxychlor, kepone), pharmaceutical agents (e.g., DES, tamoxifen, raloxifene), phthalates (e.g., di-2-ethylhexylphthalate, di-*n*-butyl phthalate), and phytoestrogens (e.g., genistein, daidzen) [1,70,71]. While there is no clear data demonstrating that environmental chemicals are the cause of reproductive anomalies in humans, the homology in organ systems between animal models and humans indicates a potential for adverse effects on sexual development and function.

Although binding affinity of xenoestrogens for ERs is low, ranging from 0.0001% to 1% of E2 levels, these chemicals have the ability to activate ERα and ERβ as agonists or prevent their binding by endogenous ligands when acting as antagonists [72,73]. Just as diverse as the number of chemicals known to exhibit estrogenic activity, the profile of biological responses to exogenous chemicals is affected by a variety of factors in reproductive tissues: animal strain and species differences, relative amounts of ER subtypes, presence of EREs, recruitment of co-regulatory proteins (co-activators and repressors), binding to plasma proteins, chirality of chemicals, and multiple mechanisms of action (e.g., estrogenicity versus antiandrogenicity) [71,74]. Specifically, xenoestrogens evoke estrogenic responses and cause their effects by mimicking and/or blocking the actions of endogenous E2 (agonist versus antagonist), and these effects may be result in changes in steroid hormone receptor gene expression, altered steroid hormone metabolism, cross-talk between ERs and other signaling systems (e.g., aryl hydrocarbon and EGF), and interference with serum protein binding [75-77]. It has also been suggested that the presence of xenoestrogens in the hormonal milieu of estrogen-sensitive tissues has the effect of potentiating E2 action [78]. The nature of the ERE in the promoter region of target genes may affect cellular response as indicated by the ability of ERβ to activate EREs from the vitellogene while ERα showed greater activation at the more divergent LH EREs in COS-1 cells [79]. Because ERs function as dimers, estrogen responsive genes may respond differently to ERα and ERβ homodimers or ERα/ERβ heterodimers following ER activation [80]. Furthermore, there are ligand-dependent differences in the ability of ERα and ERβ to bind co-regulatory proteins [21,81]. Thus, the cellular response to ER agonists and antagonists is the result of interaction between several factors.

**Figure 3:**
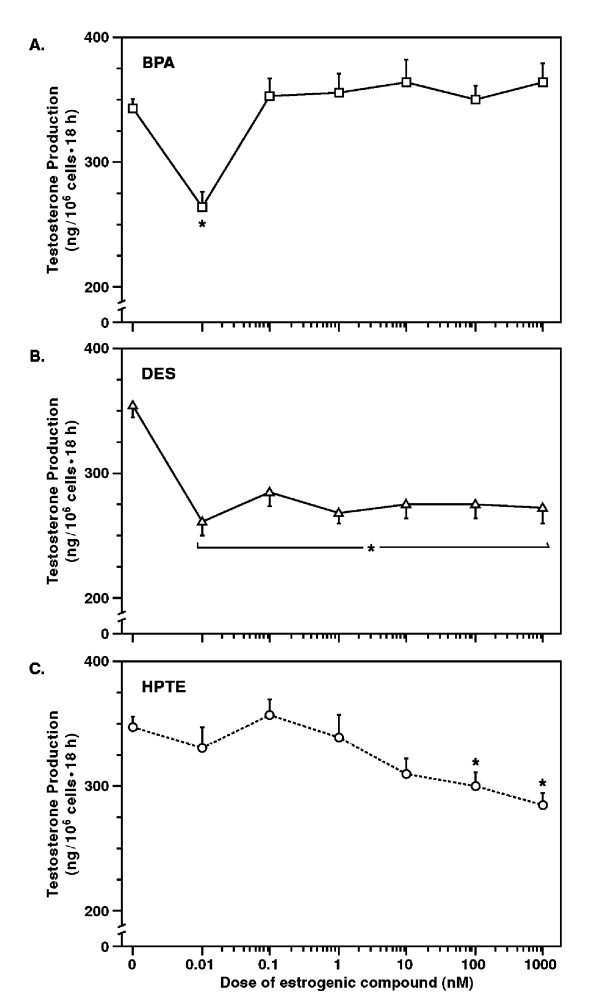
ER agonists regulate androgen biosynthesis in Leydig cells. Incubation of mature rat Leydig cells, from 90-day old rats, with estrogenic chemicals, i.e., bisphenol A (BPA)(A), the synthetic estrogen diethylstilbestrol DES (B) and a biologically active metabolite of the pesticide methoxychlor (HPTE)(C), caused an inhibitory effect on androgen biosynthesis albeit at different doses. Using RT-PCR, ERβ was not detected in these cells, implying that inhibitory effects were ERα-mediated (ref. 89). *Copyright 2004, The Endocrine Society.*

A detailed discussion of the effects of environmental chemicals on male reproduction is outside the scope of the present review and can be found elsewhere [82,83]. However, studies of estrogenic chemicals have been conducted in laboratory species with low (physiological) and high (pharmacological) doses. Data from these investigations indicate that estrogen action is dose-dependent and may be stimulatory or inhibitory. For example, exposure to E2 restored spermatogenesis to the germ cell-depleted testis of hypogonadal mice [84], decreased the rate of apoptosis and stimulated proliferation of mouse and rat spermatogonia *in vitro *[43,85], and induced renewal of spermatogonial stem cells in the testis of the *Japanese eel *[86]. On the other hand, incubation with E2 and DES was found to inhibit development of spermatogonia, Leydig cells and Sertoli cells in the fetal rat testis [87]. Administration of low doses of the industrial and estrogenic chemical bisphenol A (BPA) reduced spermatogenesis in mice [88], decreased DNA synthesis by immature rat Leydig cells (author's unpublished observations), and suppressed androgen biosynthesis by mature rat Leydig cells (Fig. 3). The effects of E2 and BPA on spermatogonial divisions and Leydig cell steroidogenesis were blocked by co-incubation with antiestrogens ICI 164384 and ICI 182,780, respectively, indicating that these effects were ER-mediated [1,89]. There is also evidence showing that E2 regulates ER gene expression in a dose-dependent manner because chronic exposures of mice to 0.5 or 50 μg/ml BPA decreased ERβ and increased ERα gene expression in germ cells [90] but a single injection of estradiol benzoate at high doses (500 μg) caused the opposite effect in prepubertal rats, i.e., decreased ERα mRNA levels and increased ERβ expression [91]. Disparities in data from different laboratories are probably due to several factors, which act to moderate estrogen signaling in sensitive tissues, e.g., interaction between transcriptional and non-transcriptional signaling pathways, receptor cross-talks, unpredictable mixture effects, and changes in steroid production and action. In addition, an inverted U-shaped dose-response, in which low doses are stimulatory and high doses are inhibitory, has been proposed for estrogen action in reproductive tissues [92].

In agreement with studies conducted in rodents, evidence supporting a direct role for estrogen in male reproductive tract development was collected from men. For example, poor semen quality has been a consistent finding in male patients with mutations in ERα [52] as well as those suffering from aromatase deficiency [53,54]. A recent study involving a large cohort of men concluded that prenatal DES exposure is associated with testicular cancer and malformations of the genitalia although fertility was not affected [93]. Increased incidence in testicular cancer was thought to be due to early life-stage exposures to environmental estrogens and/or antiandrogens, which interfere with the ability of gonadal steroids to support tissue differentiation in the fetal period [94,95]. Indeed, elevated blood estrogen levels in dizygotic twin pregnancies are known to increase the risk of testicular cancer in males [96]. Growing epidemiological evidence in support of these observations has led to the hypothesis, which states that a testicular dysgenesis syndrome (TDS) that is characterized by hypospadias, testicular cancer, abnormal spermatogenesis and undescended testis, is the result of interaction between genetic and environmental factors, including inappropriate exposures to endocrine-active chemicals [97,98]. The growing incidence of TDS in the population implies that changes in steroid hormone synthesis and action cause greater effects during sexual differentiation in humans, as in rodents, but it is not clear that sperm function and fertility are affected in adulthood.

A series of data were published lately to highlight aspects of estrogen action in the testis. First, it was observed that neonatal treatment of prepubertal rats with DES alone (0.1 μg) induced only minor effects, which were amplified after suppression of androgen production and action. Thus, it was hypothesized that reduced androgen levels render the reproductive tract more sensitive to estrogen stimulation, and that the ratio between androgen and estrogen, rather than their absolute levels, is the critical determinant of E2 action [99,100]. Curiously, this line of thinking does not explain similarities in the phenotypes of ARKO and αERKO mice, which have comparable serum androgen levels but exhibit different E2 levels (Table 2). However, there are suggestions that phenotypic similarities in ARKO and αERKO mice are possibly due to the confounding effects of growth factors that activate signaling pathways mediating E2 activity, e.g., EGF [59]. Also of interest are recent data showing that the presence of soy in the diet decreases body and testis weights, suppresses gonadotropin secretion, and retards germ cell development in the rat [101]. These findings have implications for analysis of estrogen action in the testis because: 1) The normal rat chow contains significant levels of phytoestrogens (200–300 mg/kg), potentially interfering with the action of E2 and estrogenic chemicals in reproductive tissues [102,103]; and, 2) Putative health benefits associated with soy-based diets may be confounded by phytoestrogen signaling in the testis [104]. Although there is no evidence that consumption of soy-based diets has deleterious effects on testicular function, the possibility that such effects may occur in the prepubertal period, i.e., in infanthood, cannot be discounted as this population is not routinely examined for reproductive health. Because acquisition of adult sexual behavior is dependent on priming of sexually dimorphic hypothalamic nuclei by steroid hormones in the perinatal period [105], such evaluations seem to be warranted.

## IV. Conclusion

**Figure 4:**
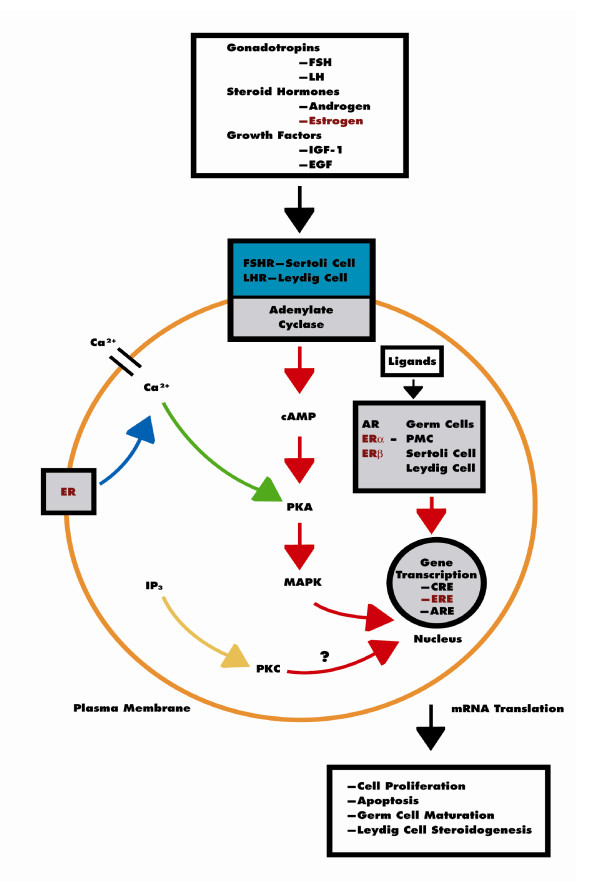
Endocrine regulation of the testis. Pituitary gonadotropins are the chief regulators of testicular function; FSH acts through its receptors in Sertoli cells (FSHR) to regulate spermatogenesis and LH stimulates androgen production by Leydig cells after binding to LHR. However, gonadal steroids, i.e., androgen and estrogen, and other agents that bind or prevent binding to steroid hormone receptors (androgen receptor AR, ERα, and ERβ), which are present in Sertoli cells, germ cells and Leydig cells also regulate testicular function. The pathway mediated by adenosine-3',5'-cyclic monophosphate (cAMP) appears to be the primary intracellular signaling pathway in all testicular cells. However, several growth factors e.g., insulin like growth factor-1 (IGF-1) and epidermal growth factor (EGF), acting via their receptors, IGF-1R and EGF-R, possibly modulate AR and ER-mediated pathways. Thus, testicular function is regulated by interactions between several signaling pathways, some acting locally, e.g., AR and ER-mediated pathways, and others indirectly by modulating hypothalamus-pituitary function. Hormonal activation of transcriptional gene activity results in changes in cell differentiation and function. PMC, peritubular myoid cell; CRE, cAMP-responsive elements, ARE, androgen-responsive elements; ERE, estrogen-responsive elements.

The major source of E2 biosynthesis is the testis in rodents and adipose tissue in men but the receptor protein (ERα and ERβ) is localized to most cell types in the testis of both species in consonance with a physiological role for estrogen in testicular development and function (Fig. 4). It is therefore not surprising that targeted deletion of the aromatase gene, ERα, and/or ERβ caused a variety of testicular anomalies in mutant mice. For example, evidence for direct ER-mediated action in testicular cells is provided by disruption of spermatogenesis in ARKO and αERKO mice and the requirement for ERα for estrogen action in Leydig cells [6,7]. Moreover, elevated serum LH levels in αERKO mice indicate that ERα deficiency jeopardizes steroid hormone negative feedback mechanisms in the hypothalamus-pituitary axis [5,106]. These observations have clinical relevance because men with disorders of glucose metabolism and those with increased body mass index (i.e., overweight or obese) exhibit elevated serum E2 levels [107] with the potential for enhanced estrogen action in the testis. However, a number of confounding variables need resolution in order to clearly identify the mechanisms associated with the physiological actions of E2 in the testis. In this regard, new experimental approaches are needed, and may include: i) use of tissue-specific knockouts in order to remove effects of concurrent ER-mediated activity in the hypothalamus and/or pituitary [108]; ii) analysis of signaling pathways not mediated by ligand binding of ERα and/or ERβ [109,110]; iii) investigation of the role of membrane ER signaling in the regulation of testicular function [111,112]; iv) assessment of the influence of genetic background on estrogen action [96,113]; v) development of methods for measuring bioavailability of estrogens in the body in order to define dose-effect relationships [114,115]; and vi) use of techniques that maintain the normal hormonal milieu of reproductive tract tissues during investigation, i.e., as related to gonadotropin and androgen action [116]. A combination of these approaches will advance our understanding of the regulatory role of E2 in the mammalian testes.

## Abbreviations

ER, estrogen receptor; E2, 17β-estradiol; DES, diethylstilbestrol; HPT, hypothalamus-pituitary-testicular axis; GnRH, gonadotropin releasing hormone; FSH, follicle stimulating hormone; LH, luteinizing hormone; ERE, estrogen response elements; IGF, insulin growth factor; EGF, epidermal growth factor; ARKO, aromatase knockout mice; αERKO, ERα knockout mice; βERKO, ERβ knockout mice, αβERKO mice, mice deficient in both ERα and ERβ; BPA, bisphenol A.
